# Changes in Th9 and Th17 lymphocytes and functional cytokines and their relationship with thyroid-stimulating hormone receptor antibodies at different stages of graves’ disease

**DOI:** 10.3389/fimmu.2022.919681

**Published:** 2022-07-22

**Authors:** Xuan Ren, Hui Chen

**Affiliations:** Department of Endocrinology and Metabolism, Lanzhou University Second Hospital, Lanzhou, China

**Keywords:** graves’ disease, TRAb, Th9 cells, Th17 cells, immunotherapy

## Abstract

**Objective:**

Graves’ disease (GD) is an organ-specific autoimmune disease characterized by the production of thyroid-stimulating antibodies (TSAb). The newly discovered CD4^+^ T helper cells, Th9 and Th17 lymphocytes, have been confirmed to be closely associated with a variety of immune diseases. However, relationships with the onset and development of GD remain unclear. The purpose of this study was to investigate the roles of Th9 and Th17 in the pathogenesis and prognosis of GD.

**Patients:**

We recruited 26 patients with newly diagnosed GD, 45 patients with GD in remission, and 20 healthy individuals.

**Measurements:**

Thyroid function and autoantibodies were evaluated using chemiluminescence immunoassays. Th9 and Th17 cells were analyzed using flow cytometry. The expression of Foxo1, IRF-4, RORc, IL-9, and IL-17 mRNA was examined using real-time PCR, and IL-9 and IL-17 protein levels were measured using enzyme-linked immunosorbent assay.

**Results:**

Th9, Th17, and characteristic cytokines IL-9 and IL-17 in the GD-untreated group were significantly higher than those in the control and remission groups. The above indexes significantly decreased in the remission group, with the levels in the TRAb^−^ remission group being similar to those in the normal group, while in the TRAb^+^ remission group, levels were differentially increased. TRAb titer was positively correlated with the levels of Th9, Th17, and their functional cytokines.

**Conclusions:**

Th9 and Th17 cells may be involved in the pathogenesis and disease outcome of GD, which could provide a new direction for developing immunotherapy for patients with GD.

## Introduction

Graves’ disease (GD) is a common organ-specific autoimmune disease characterized by diffuse goiter and thyrotoxicosis, as well as extrathyroidal manifestations, such as pretibial myxedema and Graves’ orbitopathy (GO). Elevated circulating thyroid-stimulating hormone receptor antibodies (TRAbs), especially thyroid-stimulating antibody (TSAb), are currently considered the most critical pathogenic factor for GD onset and the determinant of its development. Traditional anti-thyroid drugs, radioiodine, and surgery all have certain limitations, such as recurrence and permanent replacement therapy for thyroid hormones (1). Interruption of immune system tolerance to thyroid antigens is the immunological basis of the occurrence of GD. Specific immunotherapy for thyroid-stimulating hormone receptor has received increasing attention, and new immune interventions are expected to cure GD and avoid long-term medication for GD patients (2).

The development of GD is a complex process involving genetics, the environment, infection, and other factors. The immune imbalance caused by these factors leads to a cascade of immune responses that destroy the immune homeostasis of the body. T helper CD4^+^ lymphocytes (Th cells) play a crucial role in immune system regulation by secreting related cytokines. Classical Th cells are divided into Th1 and Th2 cells, and their imbalance is an important aspect of the pathogenesis of GD (3). Th9 and Th17 are newly discovered effector T cells, which greatly enrich the nosogenesis of immune-related diseases. Th17 cells are involved in many inflammatory, infection and autoimmune diseases, including GD (4). Th17 cells promote the occurrence and development of Hashimoto’s thyroiditis (HT) and GO, which is beyond doubt (5–7). However, its significance in GD is still unclear and controversial. High Th17 levels may be related to the refractory nature of GD (8).

In recent years, research on the differentiation and function of Th9 cells, a new type of helper T cell subset, has progressed rapidly. IL-9 is a characteristic cytokine produced by Th9 cells with multiple biological effects, including stimulating the development of autoimmune diseases, such as rheumatoid arthritis, psoriasis, and systemic lupus erythematosus, preventing parasitic infections, and inducing allergic inflammation and anti-tumor immunity (9). The important role of Th9 cells in immune regulation and antitumor activity can provide a promising therapeutic strategy (10). However, GD research has mainly involved Th1/Th2 and Th17/regulatory T cells (Tregs). Therefore, the influence of Th9 cells on the pathogenesis and progression of GD remains unclear.

In this study, we investigated Th17 and Th9 cells and the characteristic cytokines IL-17 and IL-9 in the peripheral blood of patients with GD at different stages, and we have further clarified the relationship between TRAb titer changes and Th17 and Th9 immune responses. Our research will be of certain significance to further explore the pathogenesis of GD and the prospect of new immunotherapy.

## Materials and methods

### Subjects and grouping

**Figure 1:**
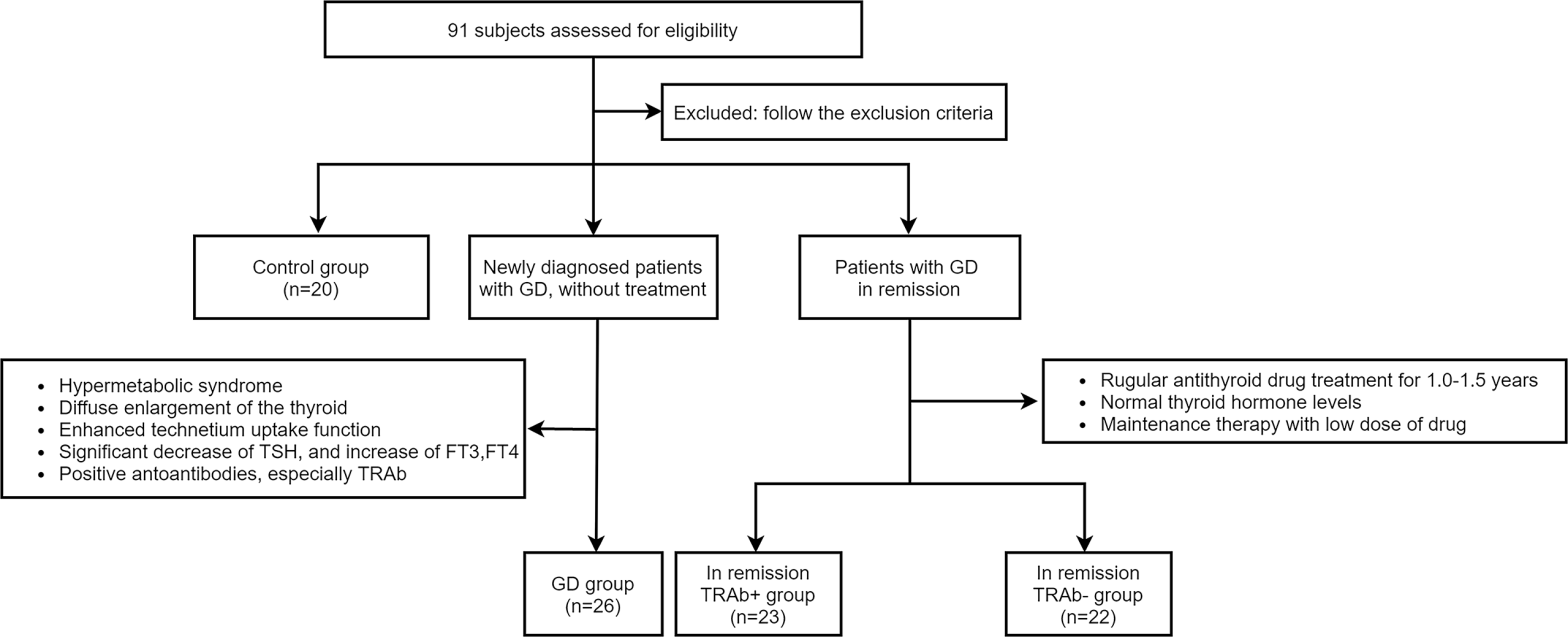
According to the inclusion and exclusion criteria, four groups were established in the experiment: Control group, Graves’ disease (GD) group, and in remission thyroid-stimulating hormone receptor antibody (TRAb^+^ and TRAb^−^) groups.

Twenty healthy individuals, 26 patients with newly diagnosed GD, and 45 patients with GD in clinical remission were recruited for this study. According to TRAb levels, 45 patients in remission were divided into two groups: 23 were TRAb-positive (TRAb^+^ in remission) and 22 were TRAb-negative (TRAb^−^ in remission). Twenty healthy age-and sex-matched individuals were used as the control group. The diagnostic criteria of newly diagnosed GD according to the 2018 European Thyroid Association Guideline for the Management of Graves’ Hyperthyroidism (11). All subjects underwent an adequate thyroid evaluation, including GD history, clinical symptoms, signs, thyroid ultrasonography, thyroid function including thyrotropin (TSH), free triiodothyronine (FT3), free thyroxine (FT4), and autoantibodies, including anti-thyroid peroxidase (TPOAb), anti-thyroglobulin (TGAb), and TRAb. Patients with acute or chronic infections, other autoimmune or chronic diseases, malignant tumors, liver and kidney insufficiency, pregnancy, breastfeeding, or those taking interferon, glucocorticoids, antibiotics, lithium carbonate, or other drugs that affect the immune system and thyroid function were excluded. The remaining subjects were included and grouped as shown in **Figure 1**. The research protocol was reviewed and approved by the Medical Ethics Committee of Lanzhou University Second Hospital, and all subjects provided informed consent.

### Thyroid function and autoantibodies

After fasting for > 8 h, whole blood samples from all subjects were collected and sent to the nuclear medicine department for thyroid serological detection. Serum concentrations of TSH (reference range: 0.38–4.34 uIU/mL), FT3 (reference range: 2.77–6.31 pmol/L), FT4 (reference range: 10.44–24.38 pmol/L), TPOAb, TGAb, and TRAb were determined using chemiluminescence immunoassay (SIEMENS, Germany). TPOAb and TGAb titers <60 U/mL and TRAb titer <1.50 IU/L were considered negative.

### Flow cytometric analysis of Th9 and Th17 cell frequencies

Peripheral blood mononuclear cells were isolated using a Ficoll density gradient. RPMI 1640 containing 10% FBS was used to dilute the cell density to 4 × 10^6^ cells/mL, 500 μL cells were resuspended in 24-well plates, and 2 μL cell activation cocktail (with brefeldin A) (BioLegend, USA) was added to each well and incubated for 5 h at 37°C in an incubator with 5% CO_2_. The cells cultured *in vitro* were centrifuged and resuspended, and PerCP-conjugated anti-human CD3 (BioLegend) and FITC-conjugated anti-human CD4 (BioLegend) antibodies were added and incubated for 15 min at 25°C in the dark. The cells were then fixed using fixation buffer for 20 min in the dark. After resuspension, fluorescently labelled PE-conjugated anti-human IL-9 (BioLegend) and APC-conjugated anti-human IL-17 (BioLegend) antibodies were added, and intracellular staining was performed for 20 min. Finally, the cells were washed, centrifuged, resuspended, and analyzed on a FACSCanto flow cytometer (BD FACSCalibur). Data were analyzed using FlowJo software. Th9 cells were defined as CD3^+^CD4^+^IL-9^+^ cells, and Th17 cells were defined as CD3^+^CD4^+^IL-17^+^ cells.

### Foxo1, IRF-4, RORc, IL-9, and IL-17 mRNA expression

Total mRNA was extracted from blood cells using Trizol reagent (Invitrogen), and cDNA was synthesized using primers and a reverse transcription kit (TaKaRa Company, Japan), according to the manufacturer’s instructions. All primers were designed and synthesized by Tsingke Tech (Xi’ an, China). Real-time PCR (RT-PCR) was performed using the SYBR Premix Ex Taq TMII kit (TaKaRa) on a common RT-PCR analyzer (Bio-Rad, USA). Beta-actin was used as the internal control gene, and the relative mRNA expression levels of Foxo1, IRF-4, RORc, IL-9, and IL-17 were normalized. The primer sequences used are listed in **Table 1**.

**Table 1:** Primers used for real-time PCR analysis.

Gene	Forward primer	Reverse primer
*Foxo1*	GGCAACCTGTCCTACGCC	GCACACGAATGAACTTGCTGT
*IRF-4*	ACTTGCCTTCACAACCGTCT	CCCGAAAGAGTCAGGAATGA
*RORc*	CGGGCCTACAATGCTGACAAC	AGGGCAATCTCATCCTCGGAA
*IL-9*	AACAAGATGCAGGAAGATCCAG	AATGCCCAAACAGAGACAAC
*IL-17*	GCTGATGGGAACGTGGACTA	AGGCCACATGGTGGACAATC
*β-actin*	GGACGGAGAGACACAAGCA	AGCACAGCCTGGATAGCAAC

### Enzyme-linked immunosorbent assay measurement of IL-9 and IL-17

The plasma of all subjects was stored at −80°C, thawed, and equilibrated at room temperature for 20 min, and the concentrations of IL-9 and IL-17 were determined using an ELISA commercial kit according to the manufacturer’s instructions (Abcam, UK).

### Statistical analysis

The data are expressed as means ± standard deviation (SD), and parameter comparison between groups was performed using one-way analysis of variance (ANOVA) and LSD *post-hoc* test. Categorical variables are expressed as percentages, and χ^2^ tests were used for comparison between groups. Linear regression analysis was used to determine correlation. All data were evaluated using GraphPad Prism v.8 Software. Values of *P*<0.05 were considered significant.

## Results

### Comparison of clinical characteristics and thyroid parameters

The four groups were matched for age and sex. Compared with the control and remission groups (TRAb^+^ and TRAb^−^ groups), FT3, FT4, TRAb, TPOAb, and TGAb in the newly diagnosed GD group were significantly increased (*P*<0.01), while TSH was significantly reduced (*P*<0.01). There were no significant differences in FT3, FT4, and TSH levels between the control and remission groups (TRAb^+^ and TRAb^−^). The TRAb level in the TRAb^+^ remission group was higher than that in the control and TRAb^−^ remission groups (*P*<0.05). Details are listed in **Table 2**.

**Table 2:** Clinical characteristics and thyroid parameters of patients with Graves’ disease (GD) and controls.

	Control	Newly diagnosed GD	In remission TRAb^+^	In remission TRAb^−^
Age (Y)	39.32 ± 14.42	37.69 ± 11.83	43.64 ± 8.38	39.38 ± 10.43
Sex (M/F)	7/13	8/18	8/15	5/17
BMI (kg/m^2)^	24.55 ± 2.00	23.62 ± 2.20	24.20 ± 2.21	24.94 ± 1.76
FT3 (pmol/L)	5.07 ± 0.67	21.32 ± 6.42^*^	5.01 ± 0.66^△^	4.97 ± 0.57^△^
FT4 (pmol/L)	14.99 ± 1.82	56.87 ± 23.13^*^	15.81 ± 2.90^△^	15.89 ± 2.15^△^
TSH (μIU/mL)	2.47 ± 1.09	0.005 ± 0.004^*^	2.01 ± 0.84^△^	1.94 ± 0.99^△^
TRAb (IU/L)	0.46 ± 0.31	12.99 ± 8.49^*^	4.99 ± 2.04^*^ ^△^	0.55 ± 0.42^△▲^
TPOAb (U/mL)	37.01 ± 9.82	971.80 ± 564.40^*^	277.20 ± 374.00^△^	142.00 ± 178.90^△^
TGAb (U/mL)	24.89 ± 9.158	250.50 ± 193.10^*^	120.40 ± 115.20^△^	50.88 ± 67.11^△^

Y, year; M, male; F, female; BMI, body mass index; FT3, free triiodothyronine; FT4, free thyroxine; TSH, thyrotropin; TRAb, thyroid-stimulating hormone receptor antibody; TPOAb, anti-thyroid peroxidase antibody; TGAb, anti-thyroglobulin antibody. Data are presented as means ± SD. Statistical significance was set at p < 0.05. ^*^P < 0.05, compared with the control group; ^△^P < 0.05, compared with the GD group; ^▲^P < 0.05, compared with the TRAb^+^ remission group.

### Changes in Th9 and Th17 cell frequencies

**Figure 2:**
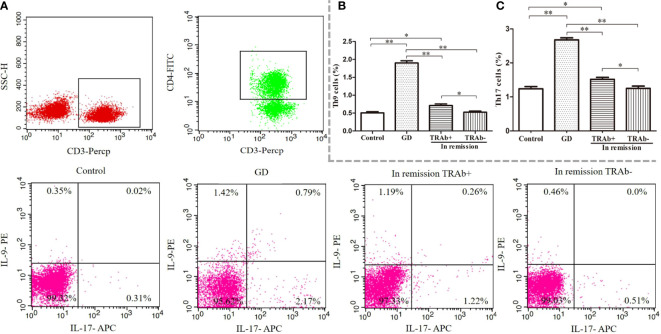
Flow cytometry analysis of Th9 and Th17 cells in patients with Graves’ disease (GD) at different stages. Gating of CD4^+^ T cell populations and intracellular cytokine staining of Th9 and Th17 cells is represented using flow cytometry for each group **(A)**. The sum of the numbers in the upper left and upper right quadrants, and the upper right and lower right quadrants is the percentage of Th9 and Th17 cell subsets in the gated CD4^+^ T cell population, respectively. Statistical analysis of Th9 and Th17 cell frequencies in the four groups is shown **(B, C)**. *P < 0.05, **P < 0.01. TRAb, thyroid-stimulating hormone receptor antibody.

Flow cytometry was used to detect the proportion of Th9 and Th17 cells in all groups, ANOVA was used to compare between groups (*P*<0.01, *F*=222.4; *P*<0.01, *F*=124.1, respectively). As shown in **Figure 2**, the proportion of Th9 and Th17 cells in the newly diagnosed GD group (1.90 ± 0.31%, 2.67 ± 0.31%, respectively) was significantly higher than that in the control group (0.50% ± 0.15%, 1.23% ± 0.28%, respectively), TRAb^+^ group (0.71 ± 0.21%, 1.51 ± 0.29%, respectively), and TRAb^−^ group (0.52 ± 0.13%, 1.25 ± 0.31%, respectively) (*P*<0.01). Compared with that in the newly diagnosed GD group, the proportion of Th9 and Th17 cells in the TRAb^+^ group was significantly decreased, but it was still significantly higher than that in the TRAb^−^ and control groups (*P*<0.05). The proportion of cells in the TRAb^−^ group was similar to that in the control group (*P*>0.05).

### mRNA expression of functional cytokines and transcription factors

**Figure 3:**
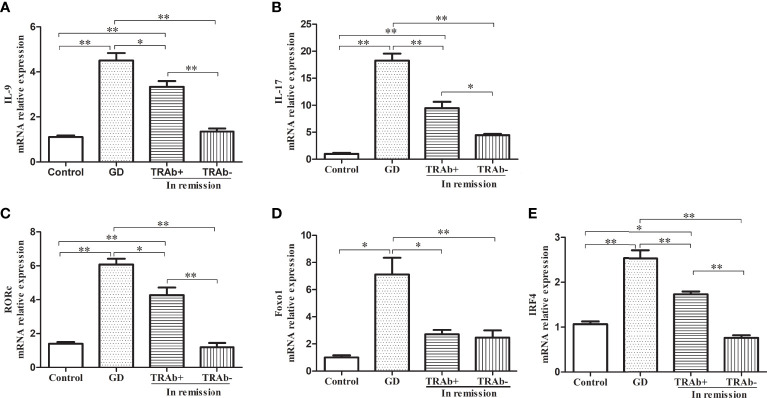
Quantitative analysis of the mRNA expression of Th9 and Th17 cell subset-related cytokines and transcription factors in patients with Graves’ disease (GD) in different stages and controls were obtained using RT-PCR. Relative mRNA expression of IL-9, IL-17, RORc, Foxo1, and IRF-4 in each group are represented by **(A–E)**, respectively. *P < 0.05, **P < 0.01. TRAb, thyroid-stimulating hormone receptor antibody.

The mRNA levels of functional cytokines IL-9, IL-17 and key transcription factors RORc, Foxo1 and IRF4 were measured and ANOVA was performed to further verify the changes of Th9 and Th17 cells at different stages of GD (*P*<0.01, *F*=53.42; *P*<0.01, *F*=71.49; *P*<0.01, *F*=56.28; *P*<0.01, *F*=14.39 and *P*<0.01, *F*=57.76, respectively). As shown in **Figure 3A**, **B**, IL-9 and IL-17 mRNA expression was significantly increased in the newly diagnosed GD group compared with that in the other three groups (*P*<0.01). IL-9 and IL-17 mRNA expression in the remission group reduced significantly, especially in the TRAb^−^ group, which was comparable to that in the control group (*P*>0.05), while mRNA expression in the TRAb^+^ group was still higher than that in the control and TRAb^−^ groups (*P*<0.05). Changes in the mRNA levels of the transcription factors RORc, Foxo1, and IRF-4 are shown in **Figure 3C–E** Consistent with the change trend in Th9 and Th17 cells and functional factors, the newly diagnosed GD group showed the highest expression of RORc, Foxo1, and IRF-4 compared with the other three groups (*P*<0.05). Both RORc and IRF-4 levels were significantly higher in the TRAb^+^ group than in the TRAb^−^ and control groups (*P*<0.05). Foxo1 did not show this trend, and there was no significant difference in its expression among the TRAb^+^, TRAb^−^, and control groups (*P*>0.05).

### Changes in IL-9 and IL-17 plasma levels

**Figure 4:**
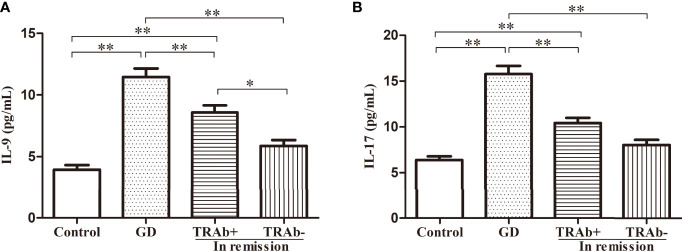
IL-9 and IL-17 plasma levels in patients with Graves’ disease (GD) at different stages and controls. *P < 0.05, **P < 0.01. TRAb, thyroid-stimulating hormone receptor antibody. Represented by **(A and B)**.

IL-9 and IL-17 plasma levels were detected using ELISA and analyzed by ANOVA (*P*<0.01, *F*=34.02; *P*<0.01, *F*=39.26, respectively). The changing trends among the four groups were consistent with the flow cytometry results and the related mRNA expression. As shown in **Figure 4**, the newly diagnosed GD patients had the highest levels of IL-9 and IL-17, which were 11.46 ± 3.51 pg/mL and 15.78 ± 4.47 pg/mL, respectively. The levels of IL-9 and IL-17 in the TRAb^+^ remission group were 8.58 ± 2.74 pg/mL and 10.42 ± 2.77 pg/mL, respectively. The lowest levels were seen in the control group (3.94 ± 1.70 pg/mL, 6.39 ± 1.80 pg/mL, respectively). Although the TRAb^−^ group level (5.87 ± 2.16 pg/mL, 8.00 ± 2.63 pg/mL) was slightly higher than that in the control group, the difference between the two groups was not significant. Compared with that in the TRAb^+^ group, the reduction in IL-9 plasma levels in the TRAb^−^ group was significant (*P*<0.05), whereas the reduction in IL-17 level was not significantly different (*P>*0.05).

### Correlations of Th9 and Th17 subsets and functional cytokines with TRAb

**Figure 5:**
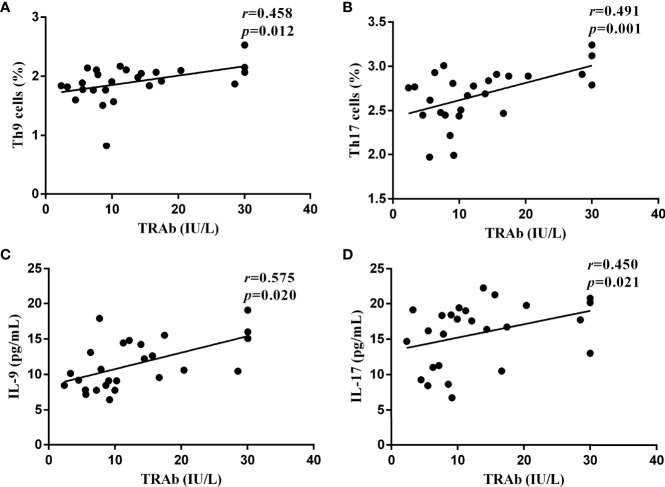
The correlations between the serological levels of TRAb and the percentages of Th9 and Th17 lymphocytes and the concentrations of IL-9 and IL-17 in newly diagnosed GD group, represented by **(A–D)**, respectively. In **(A, C, D)**, *P* < 0.05, where in b, *P* < 0.01. P < 0.05 was considered statistical difference. Represented by (A-D), respectively. In (A, C, D), P <0.05, where in B, P < 0.01.

To further confirm the role of the Th9 and Th17 lymphocytes and their functional factors IL-9 and IL-17 in the pathogenesis of GD, Pearson and Spearman correlation analyses were performed. As shown in **Figure 5**, in newly diagnosed GD group, there was a significant positive correlation between the TRAb titer and the proportions of Th9 and Th17 cells (*R*=0.458, *P*=0.012; *R*=0.491, *P*=0.001, respectively). At the same time, it was also positively correlated with the levels of IL-9 and IL-17 (*R*=0.575, *P*=0.020; *R*=0.450, *P*=0.021, respectively). However, there was no correlation between Th9/IL-9 and Th17/IL-17 levels and FT3, FT4, TSH, TPOAb, and TGAb.

## Discussion

Thyroid immune tolerance breakdown, which involves genetic susceptibility, endogenous factors, and environmental factors, is at the core of GD (12). The imbalance in immunity leads to B cell cloning, thyroid infiltration, and the production of TRAbs, mainly TSAbs, which simulate the downstream effects of TSH and TSH-R binding, leading to adenylate cyclase activation and generation of cyclic adenosine monophosphate (13). TRAbs are a class of heterogeneous polyclonal antibodies, including TSAb, thyroid stimulating blocking antibody and thyroid growth stimulating immunoglobulin (14). Although TRAb detection has been improved through electrochemiluminescence, TSAb and thyroid stimulating blocking antibody cannot be quantitatively distinguished clinically. In patients with GD, the TRAb detection level mainly comprises TSAb. TSAb production is a decisive factor in the pathogenesis and extrathyroidal manifestations of GD, and its specificity and sensitivity to GD are up to 99.0% (15). GD progression can manifest in the active, remission, and relapse phases. Changes in thyroid function are also accompanied by changes in TRAb titers. This is related to fluctuations in immune response in the body, but it is also largely affected by treatment type (15, 16). Simultaneously, the initial expression and TRAb titer changes also guide the choice and modification of clinical treatment strategies and can accurately predict the prognosis and short-term recurrence risk of GD treatment (17). TRAb persistence suggests that GD patients require ablation therapy or long-term maintenance of low-dose antithyroid drugs. High TRAb levels indicate poor prognosis (13). TRAb determination plays a decisive role in the differential diagnosis of GD, treatment choice, antithyroid drug treatment duration, prognosis evaluation, and GO clinical evaluation (18). Therefore, the latest guidelines suggest that TRAbs can be used in all stages of GD with the same value as radioactive iodine uptake and scanning in GD diagnosis (13).

CD4^+^ Th cells have been shown to play key roles in many autoimmune diseases and cancer immunotherapy (19). Increased Th17 cells and IL-17 mRNA were found in patients with HT, and enhanced immunohistochemical expression of IL-17 and IL-22 was observed in thyroid tissues. Similarly, in HT mouse models, the severity of thyroiditis in IL-17-knockout mice was reduced (20). In patients with GD and HT, increased levels of pathogenic Th17 cells were significantly associated with disease activity, presence of GO, and disease duration (21).

Novel helper T cells also have been shown to be involved in the pathogenesis of GD, but remain unclear. The classic immune imbalance in GD involves the upregulation of Th1 cells and downregulation of Th2 cells. The number of Th17 cells and the level of IL-17 are closely related to the induction of inflammation and fibrosis in systemic sclerosis and could be used as an indicator of disease activity (22). Similarly, the upregulation of Th17 cells differentiation may promote lymphocyte infiltration in the thyroid gland of GD patients (23). Th17, Th22, and the IL-17 and IL-22 cytokines were significantly increased in patients with newly diagnosed GD and were positively correlated with TSAb levels, indicating that Th17 and Th22 are involved in the pathogenesis of GD in the Chinese population (24), which was consistent with our results. However, some scholars also have proposed that the Th17 pathogenic cytokines IL-23 and IL-1β only increased in HT, but not in GD (25). The contribution of Th17 in GD remains controversial.

Surprisingly, Th17 cells were dominant in peripheral effector T cell subsets in GO patients, playing a key role in the process of GO, and were significantly positively correlated with the activity and severity of GO (5, 26). Dysregulation of the Th1/Th2 cells ratio significantly affects the onset and severity of GD, whereas changes in the frequency of Th17 cells were associated with refractory GD (23). High concentration and persistent TRAb is a marker of GD recurrence and the basis of GO onset (15). Therefore, the relationship between Th17 cells and TRAb is worthy of further investigation. Th9 cells are relatively newly described and poorly characterized cells, with IL-9 as their hallmark cytokine, with multiple functions, such as promoting immune tolerance, enhancing immune response, and anti-tumor activity (27). Elevated Th9 and IL-9 levels in the serum or tissues of patients with psoriasis and rheumatoid arthritis are associated with disease severity and the duration of inflammation (28). However, their role in GD pathogenesis and progression has not been studied. Studies have confirmed that in patients with papillary thyroid carcinoma combined with HT, IL-9 secreted by Th9 cells was abnormally higher than that in patients without HT (29). These results suggest that Th9 cells play an important role in AITD pathogenesis and are consistent with our results in patients with GD.

Th17 cells mainly secrete IL-17, as well as IL-9, although in small amounts compared to Th9 cells. Immunotherapy targeting IL-9/IL-9R may become a new approach for refractory GD and GO in the future. Therefore we divided the patients with GD into untreated and remission groups and used TRAb as a landmark index to subdivide the remission group into a positive group and a negative group, aiming to explore the role of Th17 and Th9 cells and IL-17 and IL-9 cytokines in GD pathogenesis, their changes in disease development, and their correlation with TRAb. This study will provide a basis for monitoring new immune indices and immunologically targeted therapy for GD.

We found that the proportions of Th17 and Th9 cells in the peripheral blood of patients with untreated GD were significantly higher than that in the control group. The functional cytokines IL-17 and IL-9 showed similar trends. To further verify their impact on GD outcome, the GD remission group was also included in the study. With the effective control of hyperthyroidism by antithyroid drugs, the proportions of Th9 and Th17 cells and the concentrations of functional factors obviously decreased, compared with the untreated GD group. Interestingly, when we used TRAb as a key indicator and divided the remission group into TRAb^+^ and TRAb^−^ groups, we found different conclusions. The Th17 and Th9 cells in the TRAb^−^ group returned to a lower level, which was not different from that in the control group. The changes in plasma IL-17 and IL-9 levels conformed to this rule. However, the above indices in the TRAb^+^ group were also significantly lower than those in the untreated GD group; they were still higher than those in the TRAb^−^ group (except IL-17) and the control group, and the differences were significant. These results suggest that Th17 and Th9 cells and IL-17 and IL-9 cytokines may play important roles in GD pathogenesis and are related to disease severity. At the immunological level, it has been confirmed that the recovery of thyroid function in patients with GD does not mean complete remission of immune abnormalities. Therefore, drug treatment duration is more appropriately measured through the change in TRAb, taking the negative change in TRAb as the node of withdrawal time.

The differentiation and induction of T cell subtypes require complex internal and external signal regulation. Co-stimulation with TGF-β and IL-6 initiates Th7 cell differentiation. The autocrine cytokines IL-21 and IL-23 expand differentiation and maintain the expansion. Upregulation of the specific transcription factor RORc promotes the differentiation of T cells into Th17 cells (27). RORc is an essential transcription factor for Th17 cell differentiation. RORc is highly expressed in mature Th17 cells, recruiting neutrophils to release inflammatory mediators and amplify the immune response (30). However, under RORc deficiency, the number of Th17 cells decreases, inflammatory cell recruitment and infiltration decrease, and the degree of inflammation and autoimmunity is alleviated (31). Co-stimulation with TGF-β and IL-4 can promote the differentiation of CD4^+^ T cells into Th9 cells, and IRF4 is an essential transcription factor for Th9 cell development (32). The initial T cells of IRF-4 knockout mice cannot differentiate into Th9 cells normally (10). Small interfering RNA downregulates the expression of IRF-4 and significantly inhibits IL-9 secretion by Th9 cells (33). As a representative member of the forkhead transcription factor family, Foxo1 is widely involved in a series of pathophysiological processes, such as cell proliferation, apoptosis, oxidative stress, and energy metabolism, and it maintains body homeostasis through anti-oxidation, cancer suppression, metabolism regulation, and immunity balancing (34). The regulatory role of Foxo1 in Th9 cell differentiation has only recently been clarified, and it is considered an essential transcription factor for Th9 cell development and IL-9 induction (35). Foxo1 is differentially expressed in Th9 cells (36). Foxo1 binds to and transactivates the IL-9 promoter, and IRF-4 is synergistically enhanced. In addition, Foxo1 can also bind to the IRF-4 promoter to activate IRF-4. Inhibition or deletion of Foxo1 can reduce IL-9 production by Th9 cells and significantly improve the inflammatory response in allergic asthma (35). Therefore, in addition to selecting the classic transcription factors RORc and IRF-4, we analyzed the mRNA of the newly proposed essential transcription factor Foxo1. The results showed that the mRNA levels of RORc, IRF4, and Foxo1 were significantly increased in the GD-untreated group, and RORc and IRF4 also showed a significant increase in the TRAB^+^ remission group, which was consistent with the change trends of Th9 and Th17 cells. Increased transcription factor expression may explain the increased differentiation of Th9 and Th17 cells.

As expected, TRAb is significantly associated with abnormal expression of Th17 and Th9 cells. TRAb is a reasonable indicator of immunological response to guide clinical decision making. The level of TRAb in the GD-untreated group was positively correlated with the levels of Th17 and Th9 cells and the functional factors IL-9 and IL-17. However, no such relationships were observed for TPOAb, TGAb, TSH, FT3, or FT4 in the above group. Bossowski et al. (37) also confirmed a significant positive correlation between TSAb and the percentage of CD4^+^ IL-17^+^ T cells, and TRAb was significantly positively correlated with the ratio of Th17/Treg cells in patients with initial GD. This is consistent with our conclusions that Th17 and Th9 cells play important roles in GD pathogenesis and are related to disease severity and duration. However, there are differing opinions. Some scholars believe that the pathogenesis of GD is related to a decrease in Treg cells but not to an increase in Th17 cells (38). Abnormal expression of Th9/IL-9 and Th17/IL-17 was also observed in patients with active immune thrombocytopenia and those in remission, but there was no correlation between Th9 cells, IL-9, Th17 cells, IL-17, and platelet count (39). These differences may be caused by the different types of Treg and Th17 cells, disease stages, detection methods, and genetic backgrounds (4, 40). Moreover, the immune effect of Th cells is closely related to the organ or tissue microenvironment and inflammatory sites of other Th cytokines.

In summary, patients with untreated GD exhibited high levels of Th9 and Th17 cells, and the functional factors IL-9 and IL-17 showed a consistent increase. As the disease entered the remission phase, these indicators dropped significantly. TRAb was positively correlated with Th9, Th17, and their functional factors. Our research shows that Th9, Th17, and their functional factors are involved in GD pathogenesis, and that their levels determine disease severity, treatment time, and risk of recurrence. Although the current treatments for GD are established, they are not ideal. Therefore, improved immunotherapies are still needed. Targeting the inhibition of Th9 and/or Th17 differentiation and reducing the secretion of IL-9 and/or IL-17 is a promising new approach to treating GD.

## Data availability statement

The original contributions presented in the study are included in the article/supplementary material. Further inquiries can be directed to the corresponding author.

## Ethics statement

The studies involving human participants were reviewed and approved by Medical Ethics Committee of Lanzhou University Second Hospital. The patients/participants provided their written informed consent to participate in this study.

## Author contributions

HC and XR conceived the study and its design. XR performed research and drafted the manuscript. HC supervised the research and revised the manuscript, and approved the submitted version. All authors contributed to the article and approved the submitted version.

## Funding

This study was supported by the Program for National Natural Science Foundation of China (31670518) and Gansu Provincial Science Foundation for Young Scholars of China (21JR1RA149, 21JR11RA114).

## Conflict of interest

The authors declare that the research was conducted in the absence of any commercial or financial relationships that could be construed as a potential conflict of interest.

## Publisher’s note

All claims expressed in this article are solely those of the authors and do not necessarily represent those of their affiliated organizations, or those of the publisher, the editors and the reviewers. Any product that may be evaluated in this article, or claim that may be made by its manufacturer, is not guaranteed or endorsed by the publisher.
